# ADDRESSING SOCIAL ISOLATION IN NURSING HOMES DURING COVID-19 – QUALITATIVE DATA FROM A NATIONAL SURVEY

**DOI:** 10.1093/geroni/igac059.2968

**Published:** 2022-12-20

**Authors:** Emily McPhillips, Catherine Dube, Natalia Nielsen, J Lee Hargraves, Carol Cosenza, Emily Lim, Adrita Barooah, Kate Lapane

**Affiliations:** UMass Chan Medical School, Worcester, Massachusetts, United States; UMass Chan Medical School, Worcester, Massachusetts, United States; University of Massachusetts Medical School, Worcester, Massachusetts, United States; University of Massachusetts - Boston, Boston, Massachusetts, United States; University of Massachusetts Boston, Boston, Massachusetts, United States; University of Massachusetts Boston, Boston, Massachusetts, United States; University of Massachusetts Boston, Boston, Massachusetts, United States; University of Massachusetts Chan Medical School, Worcester, Massachusetts, United States

## Abstract

The COVID-19 pandemic had dramatic, sometimes devastating impacts on nursing homes, residents, and staff. Rapid deployment of innovative approaches to resident care was required even while under sustained distress. We collected textual responses to open-ended questions about COVID-19 experiences through a national nursing home survey of Directors of Nursing/Administrators in February-May 2022. We employed a stratified (by size and quality ratings) sample of 1,669 nursing homes. Response rate was 30%, and 51% of responders answered > 1 open-ended question. We conducted an iterative thematic qualitative analysis yielding 10 themes. Respondents described addressing social isolation using new technology; enlisting staff from across the nursing home [beyond-the-call effort, gifting of voluntary time], and new ways for residents to safely connect with family. Respondents felt severely limited by COVID regulations that seemed to ignore residents’ mental health needs. The majority of respondents felt significant professional and personal impact of the pandemic experience: “The pandemic was the most stressful situation I have encountered in 26 years of nursing” – “What a toll it took on all us emotionally, physically, and mentality” – “Every day was a challenge and I felt hopeless” – Some respondents plan to quit: “I am now seeking other employment. It has been too much for too long and has directly affected my mental health.” Nursing homes reported extraordinary efforts put forth by administration and staff to meet the needs of residents. Efforts to retain nursing staff are needed given profound impacts of the pandemic on their personal and professional lives.

